# Transplantation of Nonexpanded Adipose Stromal Vascular Fraction and Platelet-Rich Plasma for Articular Cartilage Injury Treatment in Mice Model

**DOI:** 10.1155/2013/832396

**Published:** 2013-01-16

**Authors:** Phuc Van Pham, Khanh Hong-Thien Bui, Dat Quoc Ngo, Lam Tan Khuat, Ngoc Kim Phan

**Affiliations:** ^1^Laboratory of Stem Cell Research and Application, University of Science, Vietnam National University, Ho Chi Minh City, Vietnam; ^2^University of Medical Center, Ho Chi Minh University of Medicine and Pharmacy, Ho Chi Minh City, Vietnam; ^3^Department of Pathology, University of Medicine and Pharmacy, Ho Chi Minh City, Vietnam

## Abstract

Stromal vascular fraction (SVF) combined with platelet-rich plasma (PRP) is commonly used in preclinical and clinical osteoarthritis as well as articular cartilage injury treatment. However, this therapy has not carefully evaluated the safety and the efficacy. This research aims to assess the safety and the efficacy of SVF combined with PRP transplantation. Ten samples of SVFs and PRPs from donors were used in this research. About safety, we evaluate the expression of some genes related to tumor formation such as Oct-4, Nanog, SSEA3, and SSEA4 by RT-PCR, flow cytometry, and tumor formation when injected in NOD/SCID mice. About efficacy, SVF was injected with PRP into murine joint that caused joint failure. The results showed that SVFs are negative with Oct-4, Nanog, SSEA-3, and SSEA-4, as well as they cannot cause tumors in mice. SVFs combined with PRP can improve the joint regeneration in mice. These results proved that SVFs combined with PRP transplantation is a promising therapy for articular cartilage injury treatment.

## 1. Introduction

Stem cell therapy is considered as a promising therapy for degenerative disease treatment, especially articular cartilage injury as well as osteoarthritis. Osteoarthritis was treated by stem cell transplantation for a few years ago. Stem cells from various sources were used to treat this disease. However, the mesenchymal stem cells (MSCs) are considered as most suitable candidates. MSCs are multipotential cells capable of differentiation into bone, cartilage, fat, and some other cells [1]. MSCs could be isolated from bone marrow [2], adipose tissue [3], cord blood [4], banked umbilical cord blood [5], umbilical cord [6], Wharton's jelly [7], placenta [8], and pulp [9]. However, MSCs from bone marrow [10–12] and from adipose tissue [13–15] are two common stem cell sources for treating cartilage degeneration. 

Cartilage degeneration or cartilage injury is a common clinical problem and easily leads to osteoarthritis. Osteoarthritis is a chronic degenerative process characterized by the degeneration of cartilage, bone bud formation, cartilage reorganization, joint erosion, and loss of joint function [16]. Currently, cartilage injury was treated primarily with drugs [17–20] or injection of hyaluronic acid [21, 22] to reduce the symptoms, pain, and inflammation control. However, these therapies's efficiencies were limited and often failed to prevent the degeneration of the joints [23]. 

MSCs from adipose tissue, also known as stem cells isolated from fat tissue (adipose-derived stem cells—ADSCs), are a suitable source of mesenchymal stem cells for autograft. This stem cell source was used to treat many diseases such as liver fibrosis [24], sciatic nerve defects [25], systemic sclerosis [26], ischemia [27], skeletal muscle injury [28], passive chronic immune thrombocytopenia [29], and infarcted myocardium [30]. Recently, they have been extended to treat cartilage injuries as well as osteoarthritis such as dogs [31–33], rabbits [34], horses [14], rat [35], mice [36], and goats [37]. These researches demonstrated that neocartilage formed after ADSC transplantation. Some phase I and II clinical trials using ADSCs transplantation are performed to treat osteoarthritis and cartilage degeneration (NCT01300598, NCT01585857, NCT01399749). Pak (2011) showed that all ADSC grafted patients improved the cartilage regeneration [15].

Among all of ADSC transplantation cases, SVF is used as noncultured ADSC (nonexpanded ADSC). SVF transplantation has some advantages such as saving time (from isolation to transplant faster about 2-3 hours), being inexpensive, and reducing the risk of cell culture. Although many studies have demonstrated the benefits of SVF/ADSC transplantation in cartilage injury treatment, especially knee articular cartilage, so far a little comprehensive studies aim to evaluate the safety and efficiency of SVF transplantation for articular cartilage treatment. Therefore, this study aims to evaluate the safety and efficiency of SVF transplantation combined with PRP in the treatment of cartilage injury in the mouse model.

## 2. Materials and Methods

### 2.1. SVF and PRP Preparation

Firstly, adipose tissue was collected from abdominal fat tissue of ten consenting healthy donors. About 40–80 mL of fat was collected by syringe and stored in 100 mL sterile bottle. Fat was kept at 2–8°C and then quickly moved to the laboratory. SVF cells are separated from the fat using the extraction kits (Adistem, Australia) according to the manufacturer's guideline. Briefly, fat is washed 3 times with saline solution to eliminate red blood cells. Then, the fat was incubated with a solution AdiExtract (Adistem, Australia). The sample was centrifuged to collect SVF as pellet at the bottom of the tube. To prepare platelet-rich plasma (PRP), 50 mL of peripheral blood was taken from a large vein (arm veins). Blood was centrifuged 1,700 rpm for 10 minutes to get platelet-enriching plasma. This plasma is activated with activator solution (Adistem, Australia). Then, PRP was mixed with SVF to make the cell suspension. Finally, this suspension was stimulated by the LED light (light monochromatic low energy, Adlight, Adistem, Australia) for 30 minutes before using for treatment.

### 2.2. Quantification of Nucleated Cells from SVF

Cell suspension (SVF and PRP) is used to count the nucleated cells. Cell number and percentage of viable cells were determined by automatically nucleus-based cell counter (NucleoCounter, Chemometec). Total cell numbers were counted after permeabilization of the membrane by Reagent A (lysis buffer, Chemometec) and neutralized with a solution of Reagent B (Neutralized buffer, Chemometec). Cell suspension is loaded into the counting chamber containing Propidium iodide dye. For counting dead cells, suspension cells were mixed with only Reagent B solution and loaded into the counting chamber. Survival rate is calculated as follows: (total cell number − the number of dead cells): the total number of cells × 100%.

### 2.3. Evaluation of the Existence of ADSC in SVF

The existence of ADSC in SVF determined by flow cytometry. The process summarized as follows: cells were washed twice in physiological saline of Dulbecco-modified PBS (D-PBS) supplemented with 1% bovine serum albumin (Sigma-Aldrich, St Louis, MO). Cells were stained for 30 min at 4°C with the monoclonal antibody anti-CD44-PE, anti-CD90-PE, and anti-CD105-FITC (BD Biosciences, Franklin Lakes, New Jersey offers). Stained cells were analyzed by flow cytometer FACSCalibur machine (BD Biosciences). Isotype control is used for all analyzes.

### 2.4. RT-PCR

To evaluate the safety of SVF, we should identify the gene expression levels related to the process of causing tumors and test the ability to form tumors *in vivo*. About gene expression, RNA was isolate by Trizol according to the manufacturer's instructions (Sigma-Aldrich, St Louis, MO). RNA precipitated with isopropanol at room temperature for 10 minutes. ADSC cells analyzed the expression of genes related to markers of cancer cells or embryonic stem cells, Oct-3/4 and Nanog by Real-time kit SYBR RT-PCR one tube-one step (Sigma-Aldrich, St. Louis, MO). The used primers were Oct-3/4, forward primer: F: 5′-GGAGGAAGCTGACAACAATGAAA-3 ′, reverse primer R: 5′-GGCCTGCACGAGGGTTT-3; Nanog, forward primer: F: 5′-ACAACTGGCCGAAGAATAGCA-3′; reverse primer R: 5′-GGTTCCCAGTCGGGTTCAC-3; GAPDH, forward primer: F: 5′-GGGCTGCTTTTAACTCTGGT-3′; reverse primer: R: 5′-TGGCAGGTTTTTCTAGACGG-3′. 

### 2.5. *In Vivo* Tumorigenicity Assay

The tumorigenicity of ADSC was evaluated in mice NOD/SCID (NOD.CB17-Prkdcscid/J, Charles River Laboratories). All mice manipulation was according to guideline of laboratory and approved by the Local Ethics Committee of Stem Cell Research and Application, University of Science (VNU-HCM, VN). All mice were kept in clean condition. Mice were injected subcutaneously at a concentration of 10^5^, 10^6^, and 10^7^ cells, respectively in three groups (each group with 3 mice). Control group was injected with PBS. The formation of tumors in mice was followed for 3 months.

### 2.6. Articular Cartilage Injured Mice Model and Experimental Treatment Schedule

To evaluate the efficiency of SVF transplantation in articular cartilage injury, we used articular cartilage injured mouse model. The NOD mouse/SCID mice were anesthetized with ketamine (40 mg/kg), then joint destruction by fine needle 32.5 G. Normal mice were used as a positive control (uninjured). Nine mice randomly divided into the treatment group (5 mice) and negative control group (4 mice). Six hours after injury, the mice were treated. In the treatment group, 200 *μ*L containing 2 · 10^6^ SVF in PRP (the treatment group) or PBS (the negative control group) was injected into the knee joint via two doses, with a 10 min interval between injections. 

Mice were recorded some parameters related to joint regeneration for 45 days. The mice were recorded the movement on the table daily. At the 45th day, all mice were anesthetized, and their hind limbs were cut and used for histological analysis and further experiments. The samples were fixed in 10% formalin, decalcified, sectioned longitudinally, and stained with hematoxylin and eosin (HE) (Sigma-Aldrich, St Louis, MO). Using HE stained slides, three parameters were examined such as the area of injured cartilage (%), the area of neo-cartilage (%), and the number of neocartilage cell layers. The injured cartilage area was determined as the percentage of lost mature cartilage compared to the control. Data was analyzed using Statgraphics software (v7.0; Statgraphics Graphics System, Warrenton, VA).

## 3. Results and Discussion

MSCs have the large differentiative potential, easily differentiate into bone, cartilage, and adipocyte. Autologous MSC transplantation is considered as a safe and effective therapy in some patients. Recently, adipose tissue was identified as the abundant source of MSCs. ADSCs exist with large amounts of adipose tissue [38]. Similar to MSCs from other sources, ADSC have the ability to differentiate into fat cells, bone, and cartilage and transdifferentiate into neurons and muscle [39–45]. Therefore, ADSC is favored as a source of autologous cell transplantation. However, the isolated ADSC relatively complex, consuming time, so ADSCs were mainly used as SVF (containing ADSC) without culture. This study aims to evaluate the safety and efficiency of SVF transplantation resuspended in PRP in mouse model.

**Figure 1:**
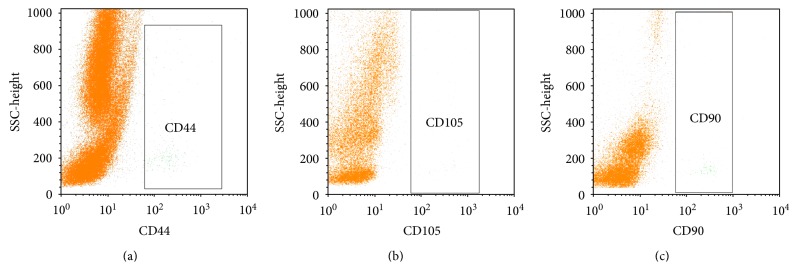
Existence of ADSCs in SVF. ADSCs were confirmed based on expression of CD44, CD105 and CD90.

In the first experiment, we successfully isolated SVF and PRP. Compared to other studies, we have successfully isolated 0.32 ± 0.15 × 10^6^ SVF cells from 1 gram of fat with a survival rate of 90.90% ± 8.57% (*n* = 10). Next, we assessed the existence of ADSC or MSC in the SVF. Analysis results from 10 samples showed that ADSCs existed in all samples. ADSC counted to 0.89% ± 0.11% in the SVF. ADSC populations were identified based on the expression of CD44, CD90, and CD105 of them (Figure 1). These results were similar to many other authors on ADSC markers [43, 46–52]. The markers satisfied the criteria of MSC following to Dominici et al. (2006) [53].

**Figure 2:**
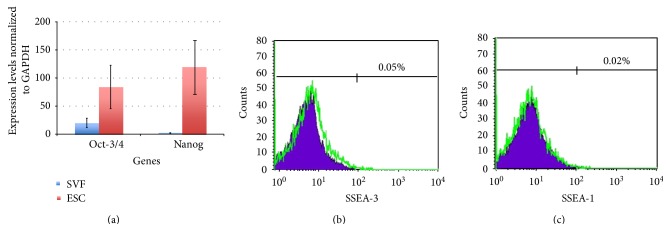
Expression of Oct-3/4, Nanog, SSEA-3, and SSEA-1 in SVF. Oct-3/4 and Nanog expressed lower in embryonic stem cell (ESC) (a); while SSEA-3 and SSEA-1 did not express in SVF (b and c).

To assess safety, we have evaluated the expression of genes related to cancer. In particular, two genes *Oct-3/4* and *Nanog *were assessed by real-time RT-PCR method and SSEA-3, and SSEA-1 was assessed by flow cytometry. The results showed expression of Oct-3/4, Nanog, SSEA-3, and SSEA-1 much lower than embryonic stem cells (Figure 2). These results demonstrated that the SVF hold low tumorigenicity. In fact, Nanog and Oct-3/4 participate in the process of self-renewal of embryonic stem cells [54, 55]. Moreover, these proteins related to the tumorigenicity process in mature germ cells [56], carcinoma oral squamous cell [57], lung cancer [58], breast cancer [59], and gliomas [60]. The SVF and PRP injection under the skin mouse NOD/SCID could not form teratomas. With these experiments, we concluded that the SVF plus PRP has a promising therapy with a high safety for transplantation experiments.

In the next experiment, we evaluated the efficiency of SVF transplantation in articular cartilage injury. The results showed that the SVF plus PRP transplantation significantly improved the articular cartilage injury compared to control. In the treated group, mice exhibited a reduction of the time required that mice could move on the table by injured hind limb compared to control. In the control group, mice can move by injured legs after 38.5 ± 4.30 days, while in the treated group, mice could move by injured legs after 29.4 ± 4.32 days.

**Figure 3:**
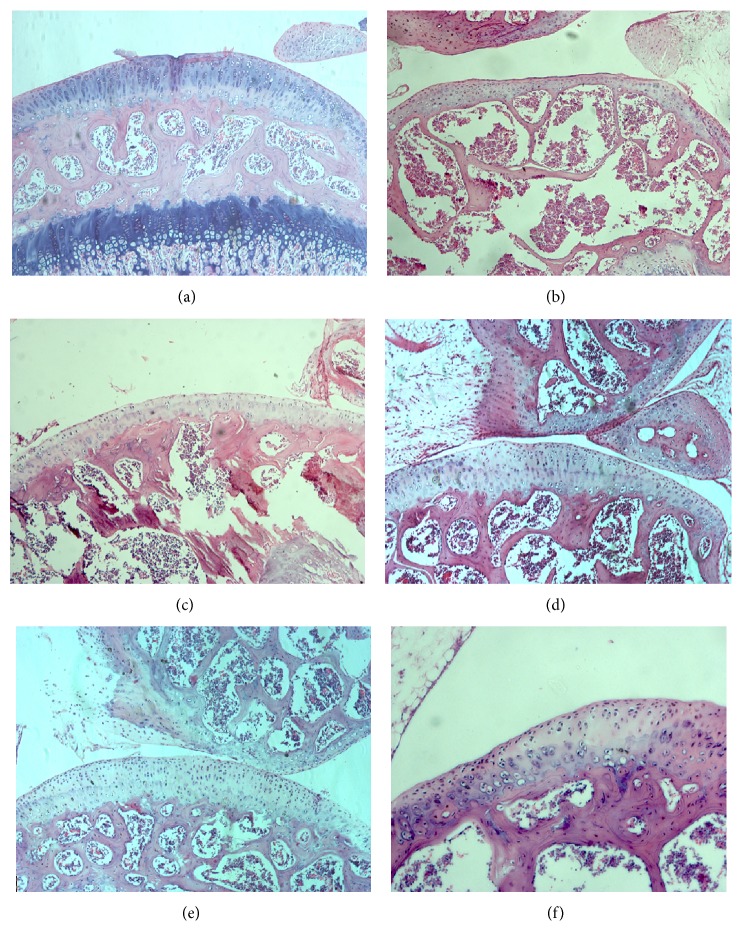
HE staining of articular cartilage. Mature cartilage layer was recorded in normal mouse (a). Mature cartilage was thinned by needle (b). Injured cartilage was regenerated in negative control group (c, e) and treated group (d, f). However, the neocartilage in treated group was thicker than in negative control group.

About histological analysis, in the treated group, an average area of the cartilage damage was 62.60%, and there was 35.5% of neocartilage formation after 45 days (*n* = 5). While in the control group, average area of cartilage lesions was 53.13%, but only 15.5% of neocartilage formation after 45 days (Figure 3).

The grade of cartilage injury between two experimental groups was different due to the effects of the dissimilar force from needle. After 45 days, results showed that 35.5% of neocartilage formed in treated group, while only 15.5% of neocartilage formed in the negative control group. This suggested that SVF and PRP gave benefit effects on the enhancement as well as trigger the neocartilage forming. More importantly, the articular cartilage in both of groups completed at the same level after 45 days with 12 cell layers. These results demonstrated that the SVF and PRP could participate in the process of self-renewal of joint cartilage at the joint microenvironment. Especially, there were no scar tissues or tumors forming at the graft sites. This result was similar to the previous publications about SVF plus PRP transplantation in the treatment of cartilage injury in dog [31–33], rabbits [34, 61], horses [14, 61], rat [35], mice [36], and goats [37]. For example, in joint injured mice model by collagenase, Ter Huurne et al. (2011) showed that the level of damage nearly 50% reduction in ADSC transplanted mice compared to control after 42 days [36]. Specifically, knee injury went down to 25% in treated mice compare to 88% in controls. They suggested that the transplanted ADSC protected and healed of injured cartilage [37]. The findings of Dragoo et al. (2007) showed that autologous ADSCs could reestablish the joint surface in rabbits, in which 100% of rabbits (12/12) had the occurrence of neocartilage, while only 8% rabbit (1/12) in the control had the appearance of neocartilage (*P* < 0.001) [62].

Roles of SVF or ADSCs in cartilage regeneration were recorded with many different effects. In fact, similar to MSCs derived from bone marrow, ADSC had anti-inflammatory properties [63, 64] and inhibition of graft versus host disease (GVHD) [65]. The transplantation of ADSC could successfully treat graft versus host disease with steroid-resistant form [66, 67]. All of these roles could add more effects to trigger rapid cartilage regeneration in this study. 

Besides, in this study, ingredients from PRP also had important roles in stimulated grafted cells as well as endogenous cells growth and differentiation. There are at least six known growth factors such as platelet-derived growth factor (PDGF) that promotes blood vessel growth, cell division, and forming the skin; transforming growth factor-beta (TGF-*β*) that promotes cell division mitosis and bone metabolism; vascular endothelial growth factor (VEGF) that promotes the blood vessel formation; epidermal growth factor (EGF) that promotes cell growth and differentiation, angiogenesis, and collagen formation; fibroblast growth factor-2 (FGF-2) that promotes the growth of cell differentiation and angiogenesis; and insulin-like growth factor (IGF) that is a regulator in all cell types of the body [68, 69]. PRP injection also showed that improvements in knee injury and osteoarthritis score, including pain and symptom relief [70, 71].

Combining effect of SVF and PRP has a positive effect on the stimulation of proliferation, differentiation, and regeneration of cartilage in a mouse model. However, SVF also has a few limitations, notably the relatively low presence of ADSCs in the SVF. Therefore, SVF cultured to enrich ADSC before being transplanted may be essential, especially a little obtained fat cases. 

## 4. Conclusion

Adipose tissue provides a rich source of MSCs. The SVF and PRP injection are a promising therapy in injured articular cartilage regeneration. This therapy significantly improved the injured articular cartilage. However, this study only assesses the ability of tumorigenicity and efficiency in mouse. Some side effects such as fever and muscle pain as well as the tumorigenicity in human being when using SVF and PRP could not be checked in this research. 

## Abbreviations

SVF: Stromal vascular fraction
PRP: Platelet-rich plasma
RT-PCR: Reverse transcription polymerase chain reaction
NOD/SCID: Nonobese diabetic/severe combined immunodeficient
SSEA: Stage-specific embryonic antigen
MSC: Mesenchymal stem cell
ADSC: Adipose-derived stem cell
D-PBS: Dulbecco-modified phosphate buffered saline
HE: Hematoxylin and eosin
ESC: Embryonic stem cell
GVHD: Graft versus host disease.
